# Rural and urban differences in undersupply of buprenorphine provider availability in the United States, 2018

**DOI:** 10.1186/s13722-021-00282-2

**Published:** 2022-01-31

**Authors:** Kevin P. Conway, Dalia Khoury, Rainer Hilscher, Arnie P. Aldridge, Stephanie J. Parker, Gary A. Zarkin

**Affiliations:** 1 National Institute of Mental Health, Bethesda, MD USA; 2 Research Triangle Institute International, Research Triangle Park, NC USA

**Keywords:** Buprenorphine, Treatment access, Opioids, Medication for opioid use disorder

## Abstract

**Background:**

Medications to treat opioid use disorder (OUD) including buprenorphine products are evidence-based and cost-effective tools for combating the opioid crisis. However, limited availability to buprenorphine is pervasive in the United States (US) and may serve to exacerbate the deadly epidemic. Although prior research points to rural counties as especially needy of strategies that improve buprenorphine availability, it is important to investigate the availability of waivered providers according to treatment need as defined by the county-level rate of opioid-overdose deaths (OOD). This study examined differences in buprenorphine provider availability relative to treatment need among rural and urban counties in the US.

**Methods:**

Buprenorphine provider availability relative to need in each county was defined as the number of waivered providers divided by the rate of OODs (i.e., number of OODs/100,000 population), according to 2018 data. Counties with ratios in the bottom tertile of their state were classified as buprenorphine undersupplied. We estimated logit models to statistically test the association of rurality and state main effects and their interaction terms (independent variables) and the county classified as buprenorphine undersupplied (dependent variable).

**Results:**

A total of 38 states and 2595 counties had sufficient non-suppressed data to remain in the analysis. A larger percent of urban counties (36.43%) than rural counties (32.01%) were classified as buprenorphine undersupplied (p  = 0.001). The likelihood of a rural county being undersupplied varied considerably by state (Chi Square  = 82.88, p  = 0.000). All states with significant (p  < 0.05 or p  < 0.10) interaction terms showed lower likelihood of buprenorphine undersupply in rural counties.

**Conclusions:**

The rural–urban distribution in undersupply of waivered buprenorphine providers relative to need varied markedly by state. Strategies for improving access to buprenorphine-waivered providers should be state-centric and informed by county-specific indicators of need.

## Background

The United States (US) continues to battle an opioid crisis that is constantly shifting and may be worsened by Coronavirus disease 2019 (COVID-19) [1, 2]. Recent national data estimate that 49,860 Americans died from an opioid-related overdose in 2019 (~ 137 per day), fueled largely by the use of synthetic opioids such as illicitly manufactured fentanyl [3]. Medications to treat opioid use disorder (OUD) including buprenorphine products approved by the Food and Drug Administration (FDA) are evidence-based and cost-effective tools for combating the opioid crisis [4–6]. However, limited availability to buprenorphine is pervasive in the US and may serve to exacerbate the deadly epidemic.

Under the Drug Addiction Treatment Act of 2000, the FDA requires physicians and other healthcare providers to obtain waivers to prescribe buprenorphine [7]. Studies show that availability to buprenorphine-waivered providers is especially limited in rural counties. For example, Rosenblatt et al. [8] reported that most physicians with a DEA waiver practice in urban counties, and Andrilla et al. [9] found that only 44% of rural counties across the US have a waivered provider. Similarly, Dick et al. [10] reported that 29.8% of rural residents live in a county without a single waivered provider, compared with 2.2% of residents living in urban counties. Although the number of waivered providers has increased over time in areas with elevated risk of opioid-overdose deaths (OODs) [10, 11], the rate of growth in nonmetropolitan and rural counties lags markedly behind urban counties [11]. Results from these studies point to nonurban counties as especially needy of strategies that improve buprenorphine availability.

Beyond the presence of buprenorphine-waivered providers by urbanicity (or rurality), it is important to investigate the availability of waivered providers according to treatment need as defined by the county-level OOD rate. Taking such an approach, Haffajee et al. [12] reported that rural counties had waivered provider rates (in 2017) that were similar to the national rate (pooled from 2015 to 2017), perhaps due to an increase in OODs in urban areas during the study period. Although this study casts doubt on rurality as a risk factor for unmet treatment need, it investigated main effects (of urbanicity) without examining potential differences between urban and rural counties within and across states. Given the observation that OODs vary considerably by county even within specific states [3] and the vital role that individual states and local governments play in combating the opioid crisis, it is critical to examine within-state patterns of buprenorphine availability relative to need to determine whether there is sufficient capacity to address the opioid epidemic locally. The purpose of this study is to examine rural–urban differences in buprenorphine provider availability relative to treatment need across states and counties in the US.

## Methods

We obtained 2018 mortality data from the Multiple Cause of Death database from Centers for Disease Control and Prevention (CDC) WONDER [3]. Drug overdose deaths were classified using the 10th revision of International Classification of Diseases, based on the underlying cause-of-death codes X40–X44 (unintentional), X60–X64 (suicide), X85 (homicide), or Y10–Y14 (undetermined intent). Deaths with the following codes were considered OODs: opium (T40.0); heroin (T40.1); natural and semisynthetic opioids (T40.2); methadone (T40.3); synthetic opioids other than methadone (T40.4); and other unspecified narcotics (T40.6). For counties with suppressed OOD counts (i.e., fewer than 10; N  = 2400), we imputed counts by summing across the non-suppressed counties within a state, subtracting the non-suppressed total from the state’s overall total (obtained from CDC WONDER published tables), and dividing the difference by the number of suppressed counties in the state.

Information on buprenorphine-waivered prescribers came from the 2018 Drug Enforcement Administration’s (DEA) Active Controlled Substances Act Registrants database. Buprenorphine provider availability relative to need in each county was defined as the number of waivered providers divided by the rate of OODs (i.e., number of OODs/1,00,000 population) in 2018. Counties with ratios in the bottom tertile of their state were classified as buprenorphine undersupplied. We classified each county as rural (micropolitan and non-core) or urban using US census definitions.

Exclusion criteria for states included having fewer than 5 non-suppressed counties (Alaska, Delaware, District of Columbia, Hawaii, Idaho, Iowa, Montana, South Dakota, and Wyoming) and lacking rural counties (New Jersey and Rhode Island) as defind above. We further excluded Maryland and Connecticut from the statistical model due to perfect prediction in the model. A total of 38 states and 2595 counties remained in the analysis.

To statistically test the association of rurality and state (independent variables) and the county classified as buprenorphine undersupplied (dependent variable), we estimated a logit model that included a county’s rural status, individual state indicators, and interaction terms indicating whether the county is in a given state and is also a rural county.

## Results

**Fig. 1:**
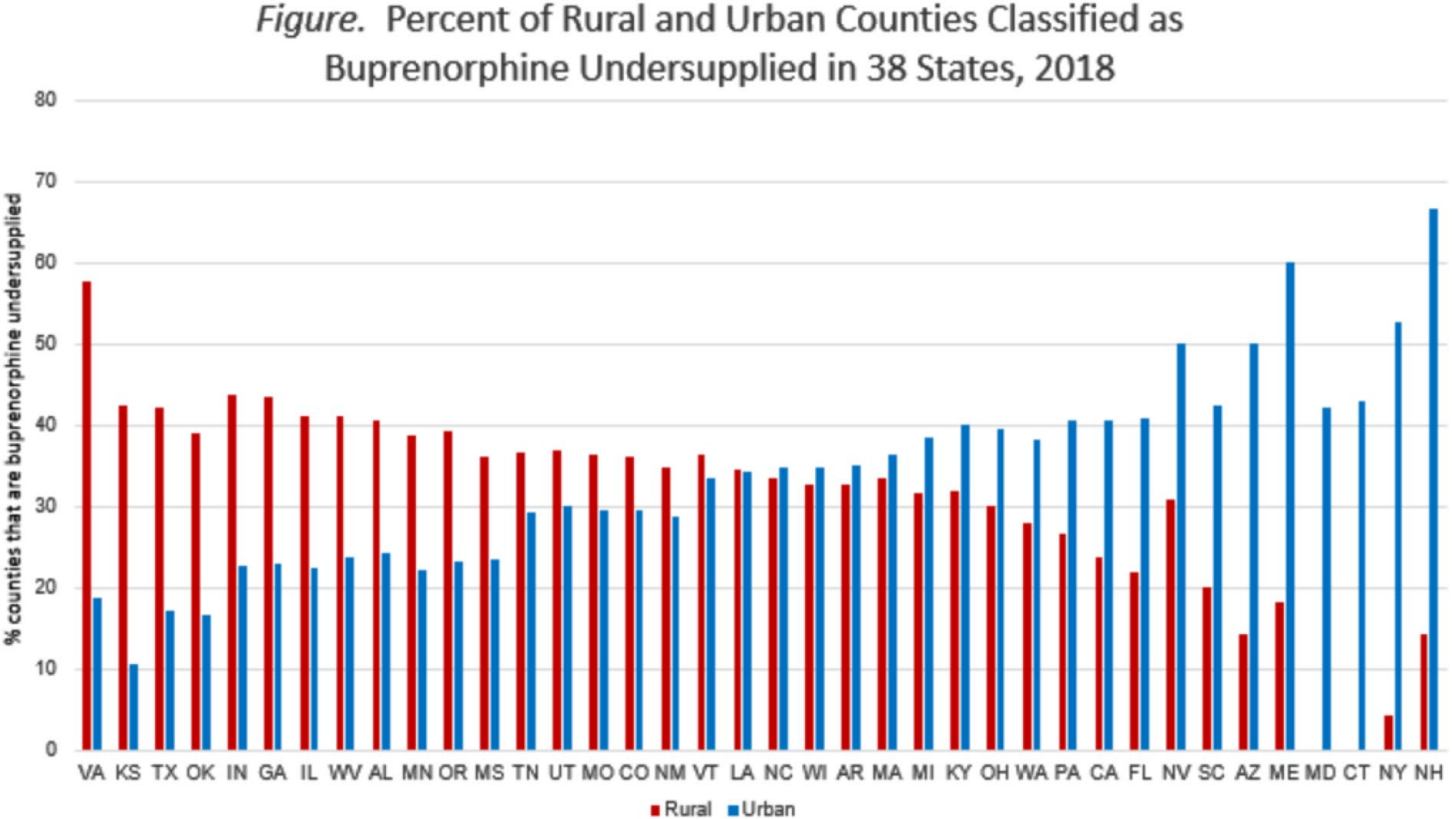
Percent of rural and urban counties classified as buprenorphine undersupplied in 38 states, 2018

For the 2595 counties in the analysis, the rate of buprenorphine providers (per 1,00,000 population) averaged 1.4 (SD  = 2.6) and ranged from 0 to 39.1. The rate of OODs (per 1,00,000 population) averaged 17.98 (SD  = 24.96) and ranged from 0.29 to 487.37; the number of OODs averaged 15.31 (SD  = 50.26 and ranged from 0.61 to 1007. The average rate of buprenorphine provider availability relative to need was 13.9 (SD  = 24.7) and ranged from 0 to 288.7. A larger percent of urban counties (36.43%) than rural counties (32.01%) were classified as buprenorphine undersupplied (p  = 0.001). The Fig. 1 displays the percentage of a state’s rural (in red) and urban (in blue) counties that were buprenorphine undersupplied in 2018. Across the 38 states, undersupply concentrated in rural counties in roughly half of the states and in urban counties in the remaining half.

The Table 1 shows the results of the logit model. Being a rural county was not systematically associated with buprenorphine undersupply (p  = 0.164) across the 38 states. We rejected the null hypothesis of equal state-rural interactions (p  < 0.000), indicating that the likelihood of a rural county being buprenorphine undersupplied varied significantly by state. Inspection of the state-by-rural interaction results shows that all four states with significant (p  < 0.05) interaction terms showed lower likelihood of buprenorphine undersupply in rural counties. For example, in Florida, the odds ratio (OR) for the interaction term (0.187, p  = 0.038) indicates that rural counties (compared to urban counties) were significantly less likely to be buprenorphine undersupplied. Specifically, rural counties in Florida were 81.3% less likely than urban counties to be buprenorphine undersupplied. For the remaining three states, this difference was 98.2% in New York, 93.1% in Maine, and 84.1% in South Carolina. Five additional states with borderline significant (p  < 0.10) interaction terms also showed lower likelihood of buprenorphine undersupply in rural counties. The difference was 92.2% in Arizona, 78.6% in California, 96.1% in New Hampshire, 69.3% in Ohio, and 75.1% in Pennsylvania.

**Table 1:** Model estimates for being a buprehorphine undersupply county in 38 states, 2018

Covariates	All states—linear probability model					
	Main effects		p value	Interactions of state × rural		
	Odds ratio	95% CI		Odds ratio	95% CI	p value
Constant	0.318***	0.136–0.745	0.008			
Rural	2.143	0.732–6.275	0.164			
AZ	3.143	0.618–15.983	0.168	0.078*	0.005–1.216	0.069
AR	1.692	0.484–5.920	0.410	0.422	0.092–1.932	0.266
CA	2.143	0.732–6.275	0.164	0.214*	0.043–1.071	0.061
CO	1.310	0.341–5.033	0.695	0.635	0.127–3.180	0.580
FL	2.176	0.768–6.166	0.144	0.187**	0.039–0.909	0.038
GA	0.937	0.342–2.570	0.900	1.206	0.336–4.327	0.774
IL	0.912	0.295–2.822	0.874	1.116	0.275–4.536	0.878
IN	0.924	0.306–2.791	0.889	1.234	0.302–5.035	0.769
KS	0.370	0.068–2.013	0.250	2.914	0.450–18.860	0.262
KY	2.095	0.707–6.212	0.182	0.326	0.085–1.256	0.103
LA	1.640	0.546–4.928	0.378	0.471	0.106–2.095	0.323
ME	4.714	0.650–34.194	0.125	0.069**	0.005–0.921	0.043
MA	1.796	0.403–8.004	0.443	0.408	0.022–7.445	0.545
MI	1.964	0.615–6.273	0.254	0.345	0.081–1.463	0.149
MN	0.898	0.259–3.115	0.865	1.027	0.228–4.620	0.973
MS	0.967	0.237–3.950	0.963	0.851	0.166–4.362	0.846
MO	1.310	0.425–4.038	0.639	0.637	0.160–2.534	0.522
NV	3.143	0.371–26.632	0.294	0.207	0.017–2.596	0.222
NH	6.286	0.492–80.274	0.157	0.039*	0.001–1.138	0.059
NM	1.257	0.198–7.976	0.808	0.618	0.074–5.150	0.656
NY	3.492**	1.207–10.107	0.021	0.018***	0.002–0.193	0.001
NC	1.676	0.590–4.766	0.333	0.437	0.113–1.700	0.233
OH	2.050	0.702–5.981	0.189	0.307*	0.076–1.236	0.097
OK	0.629	0.140–2.827	0.545	1.491	0.266–8.342	0.650
OR	0.943	0.201–4.423	0.941	1.000	0.153–6.531	1.000
PA	2.143	0.732–6.275	0.164	0.249*	0.056–1.112	0.069
SC	2.305	0.728–7.300	0.156	0.159**	0.028–0.889	0.036
TN	1.300	0.440–3.847	0.635	0.649	0.162–2.601	0.542
TX	0.647	0.232–1.807	0.406	1.653	0.470–5.810	0.433
UT	1.347	0.272–6.658	0.715	0.635	0.089–4.523	0.650
VT	1.571	0.123–20.069	0.728	0.533	0.029–9.724	0.671
VA	0.725	0.262–2.010	0.537	2.758	0.728–10.441	0.135
WA	1.934	0.568–6.581	0.291	0.292	0.052–1.646	0.163
WV	0.982	0.263–3.663	0.979	1.045	0.207–5.291	0.957
WI	1.664	0.515–5.379	0.395	0.427	0.097–1.872	0.259
Observations	2595			2595		
R-squared	0.004			0.041		
Joint Chi-squared test of equality all rural × state coefficients				82.88		0.000
Joint Chi-squared test of equality of rural × state coefficients for 10 states with the highest per capita fatal opioid overdose rate (KY, MA, ME, MO, NH, NM, OH, PA, TN, and WV)				10.36		0.322

All models use Robust Standard Errors

AL is the reference state

***p  < 0.01, **p  < 0.05, *p  < 0.1

## Conclusions

Previous studies identified rurality as a common risk factor for the undersupply of waivered providers for the treatment of OUD [8–10]. In contrast, the current study shows that the rural–urban distribution in undersupply of waivered buprenorphine providers relative to need varied markedly by state. Further, our results show that, in states with significant rural–urban differences, buprenorphine undersupply relative to need was *less* likely in rural counties. The lower likelihood of buprenorphine undersupply in rural counties was considerable and ranged from 81.3 to 98.2% (at the p  < 0.05 level) and from 69.3 to 96.1% (at the p  < 0.10 level).

Our study has limitations. There is no standard measure of buprenorphine undersupply, and our measure is relative to treatment need as defined by OOD rate within each county. Further, the DEA data on buprenorphine-waivered prescribers do not inform about the actual provision of buprenorphine, including whether providers are actively prescribing at or below their capacity. Indeed, many providers do not prescribe up to capacity [13, 14] due to a variety of reasons that are both pragmatic (e.g., time, reimbursement) and attitudinal (e.g., beliefs about agonist treatment) [15]. The DEA data also do not provide information on the quality of care. Additionally, athough we used the most recent data for buprenorphine-waivered prescribers, they remain somewhat outdated. A substantial proportion of counties had suppressed OOD data requiring imputation that may undercount or overcount OODs. Exclusion criteria reduced our sample to 38 states, thereby limiting generalizability. Finally, more research is needed on ways to increase buprenorphine provision for the treatment of OUDs, and whether doing so impacts the opioid epidemic.

In sum, our findings suggest that actions and reallocation of resources to improve availability of buprenorphine to treat OUDs must be done on a state-by-state basis that accounts for county-specific indicators of availability relative to need. To the extent that COVID-19 has exacerbated the opioid crisis [2] and will disproportionately impact individuals with OUD by interrupting availability to medication for OUD [1], coordinated state-county action is urgently warranted.

## Data Availability

Data and materials can be made available by contacting corresponding author (DK).

## Abbreviations

CDC WONDER: Centers for Disease Control and Prevention Wide-ranging ONline Data for Epidemiologic Research
COVID-19: Coronavirus disease 2019
DEA: Drug Enforcement Administration
FDA: Food and Drug Administration
OUD: Opioid use disorder
OOD: Opioid overdose deaths
